# Supplementary material for: Regulatory controls of duplicated gene expression during fiber development in allotetraploid cotton
Source: Nat Genet. 2023 Oct 16;55(11):1987–97. doi: 10.1038/s41588-023-01530-8 (PMC10632151; doi:10.1038/s41588-023-01530-8)
Supplement: Supplementary file 1 — Supplementary Figs. 1–18, Results and Methods. [file 41588_2023_1530_MOESM1_ESM.pdf]

nature genetics

Article

https://doi.org/10.1038/s41588-023-01530-8

Regulatory controls of duplicated gene expression during fiber development in allotetraploid cotton

In the format provided by the authors and unedited

SUPPLEMENTARY INFORMATION

Contents

Supplementary Figures 3
Supplementary Fig. 1 3
Supplementary Fig. 2 4
Supplementary Fig. 3 5
Supplementary Fig. 4 6
Supplementary Fig. 5 7
Supplementary Fig. 6 8
Supplementary Fig. 7 9
Supplementary Fig. 8 10
Supplementary Fig. 9 11
Supplementary Fig. 10 12
Supplementary Fig. 11 13
Supplementary Fig. 12 14
Supplementary Fig. 13 15
Supplementary Fig. 14 16
Supplementary Fig. 15 17
Supplementary Fig. 16 18
Supplementary Fig. 17 19
Supplementary Fig. 18 20
Supplementary Results 21
Functional mechanisms of *cis*- and *trans*-eQTLs 21
GWAS QTL verification using F2 population 21
GhMYB mutants show short fiber phenotypes 22
Characteristics of genetic modules 23
Genetic regulation across subgenomes 24
Co-expression network and dynamic expression bias of homoeologs 24
Supplementary Methods 26
Mutants construction and RNA-seq analysis 26
Annotation of functional variants 26
Identification of favorable expression pattern 26

Identification of co-expression clusters with dynamic expression bias 27
Identification of pseudo-regulatory sites in the other subgenome 28
Genotype and GWAS analysis for F_2 population 29
Phylogenetic analysis 29
Construction of favorable allele library 29
References 31

Supplementary Figures

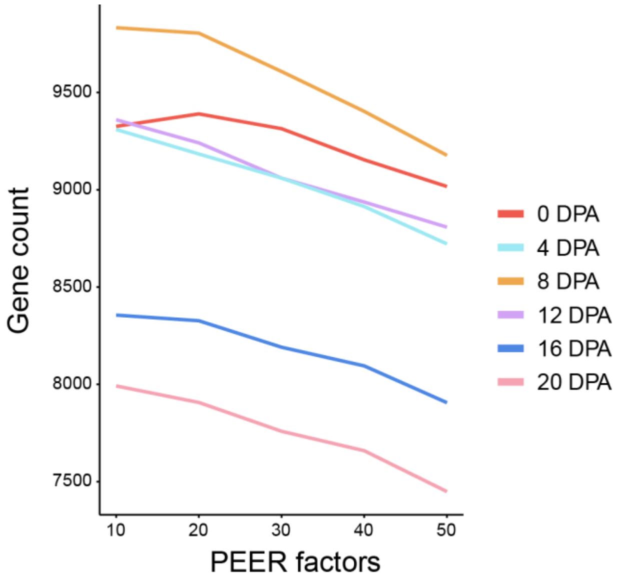

Supplementary Fig. 1: Latent factor analysis of gene expression. In each timepoint, the hidden factors with different numbers estimated by PEER are used for eQTL analysis, and the number of significant *cis*-eQTL is counted.

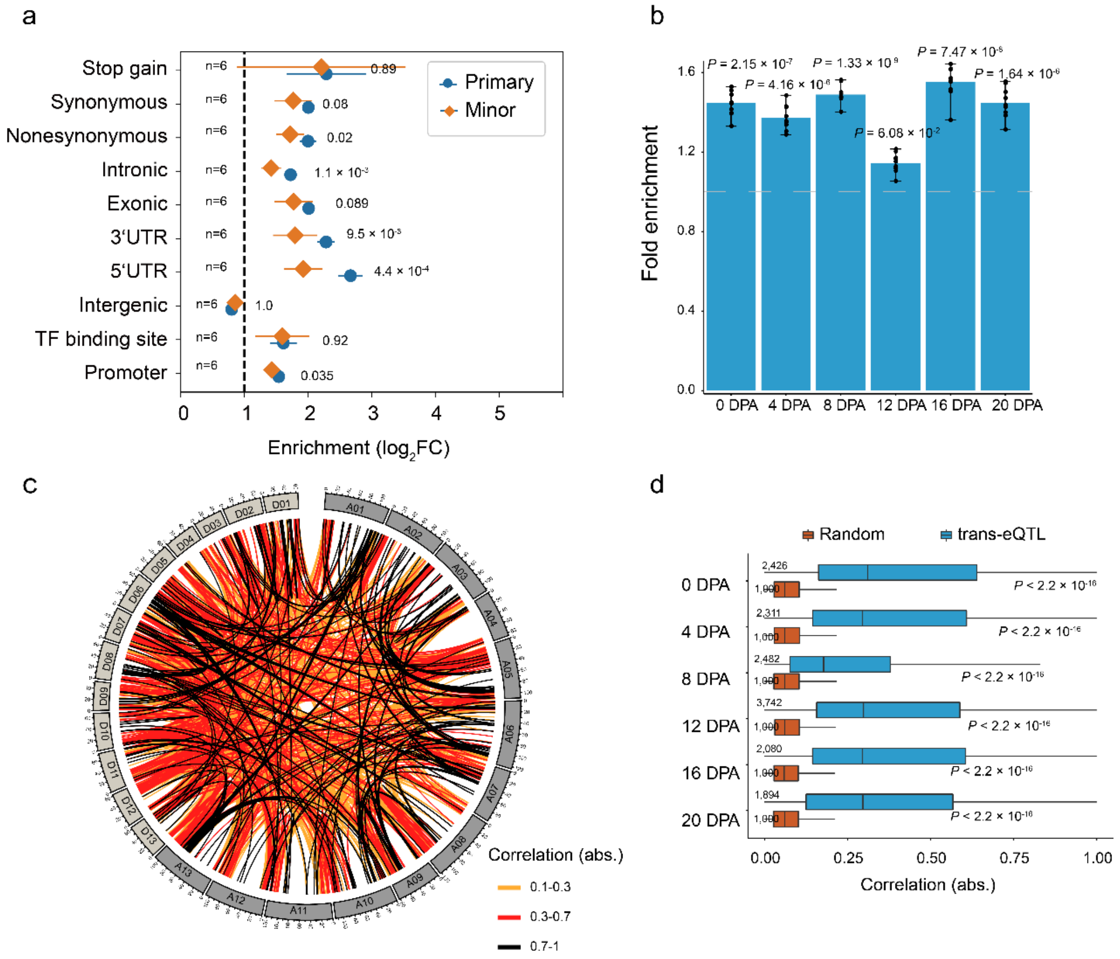

Supplementary Fig. 2: Annotation and genetic effect of functional variants. a, Enrichment of lead eVariants is shown as mean and ± SD across timepoints (n = 6 timepoints examined over 10 genomic regions; one-sided Fisher's exact test). Promoter region is defined as 2 Kb upstream of the transcription start site (TSS). **b**, Enrichment of lead *trans*-variants that have been tested for *cis*-eQTL analysis and showed significant genetic effects at the same timepoint (n = 1,000 replications were run across timepoints; one-sided Fisher's exact test; error bar, mean ± SD). **c**, Linkage disequilibrium (LD) between *cis*-eQTLs and *trans*-eQTLs of the same gene. **d**, Comparing the LD of *cis* and *trans* eQTLs regulating the same gene with that of *cis* and random SNPs. Two-sided Wilcoxon rank sum test; center line, median; box limits, first and third quartiles; whisker, 1.5 ×interquartile range.

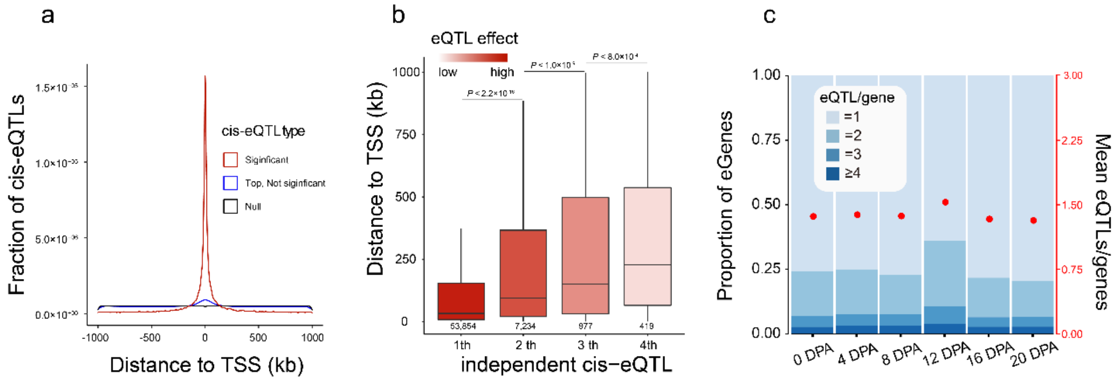

Supplementary Fig. 3: Distribution and regulatory effect of conditional eQTL per gene across fiber development. a, Distribution of cis-eQTL around TSS (1 Mb up- and downstream) at all stages. All gene-variant pairs tested as null; Significant indicates the top variant for significant eQTL of genes; Top, non-significant indicates the top associated SNP for non-significant genes. **b**, Distance to the TSS increases from the first to the fourth conditionally independent cis-eQTL (n = 53,854, 7,234, 977, and 419 cis-eQTLs; two-sided Wilcoxon rank sum test; center line, median; box limits, first and third quartiles; whisker, 1.5 ×interquartile range). **c**, Distribution and average number of eQTLs per gene across stages.

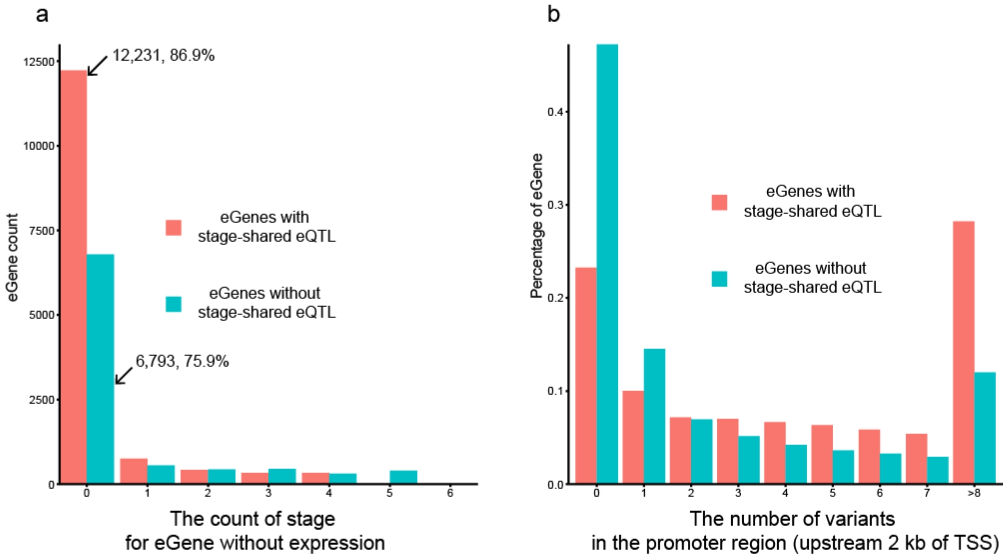

Supplementary Fig. 4: The comparison of gene expression pattern and the number of variants in the promoter region. **a**, Comparison of the stage count distribution for eGene without expression. **b**, Distribution of the count of variants in the promoter region (2 Kb upstream of the transcription start site (TSS)).

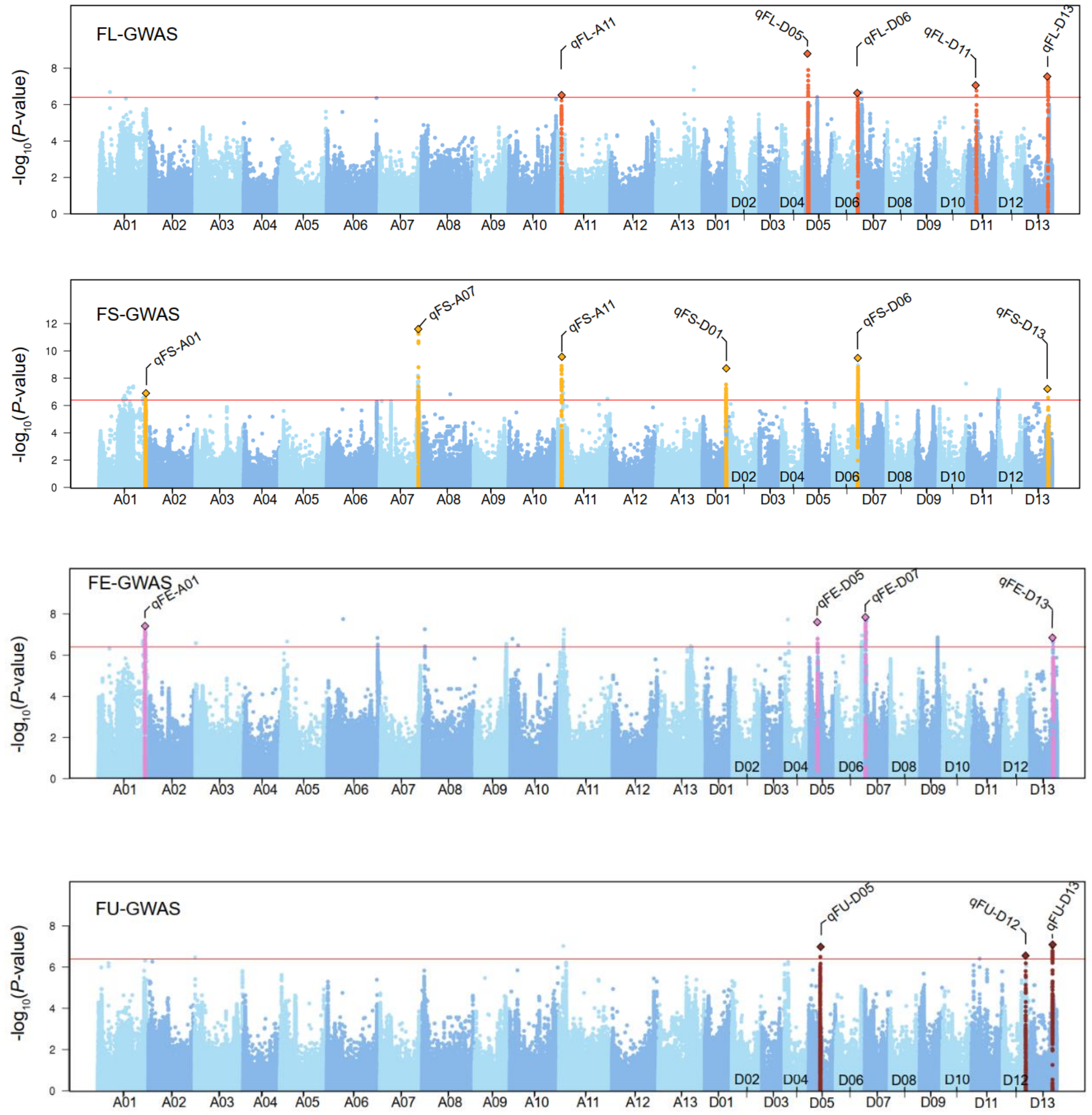

Supplementary Fig. 5: Manhattan plots of the genome-wide association study with four fiber-related traits, respectively. FL, fiber length; FS, fiber strength; FE, fiber elongation rate; FU, fiber uniformity. Significant QTLs are labeled. Significance threshold of $P = 4.0 \times 10^{-7}$ (1/n) (one-sided F-test).

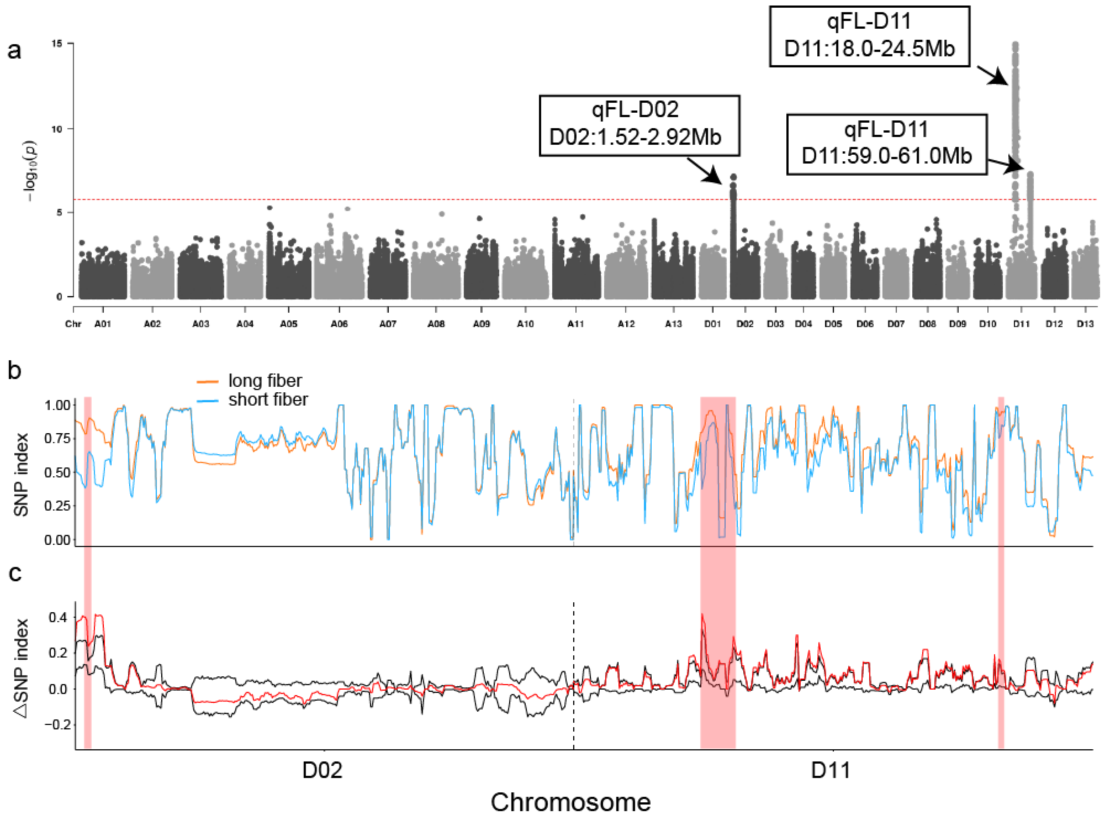

Supplementary Fig. 6: Identification of regulatory region controlling fiber length by using a segregating F_2 population. a, GWAS analysis was performed on fiber length trait using 637,372 high quality single nucleotide polymorphisms (SNPs) in 200 F_2 samples. The horizontal red line shows the significance threshold of GWAS (1/n), the x-axes show the 26 chromosomes (A01-A13 and D01-D13) in TM-1. **b**, The SNP index indicates the ratio of SNPs that are identical to those in the long-fiber parent. The long fiber pool and short fiber pool are shown in orange and blue, respectively. **c**, The SNP index (SNP index of long fiber pool subtracted that of short fiber pool) and its 95% confidence interval are shown in red and black lines, respectively. The identified QTL regions are highlighted with pink bars.

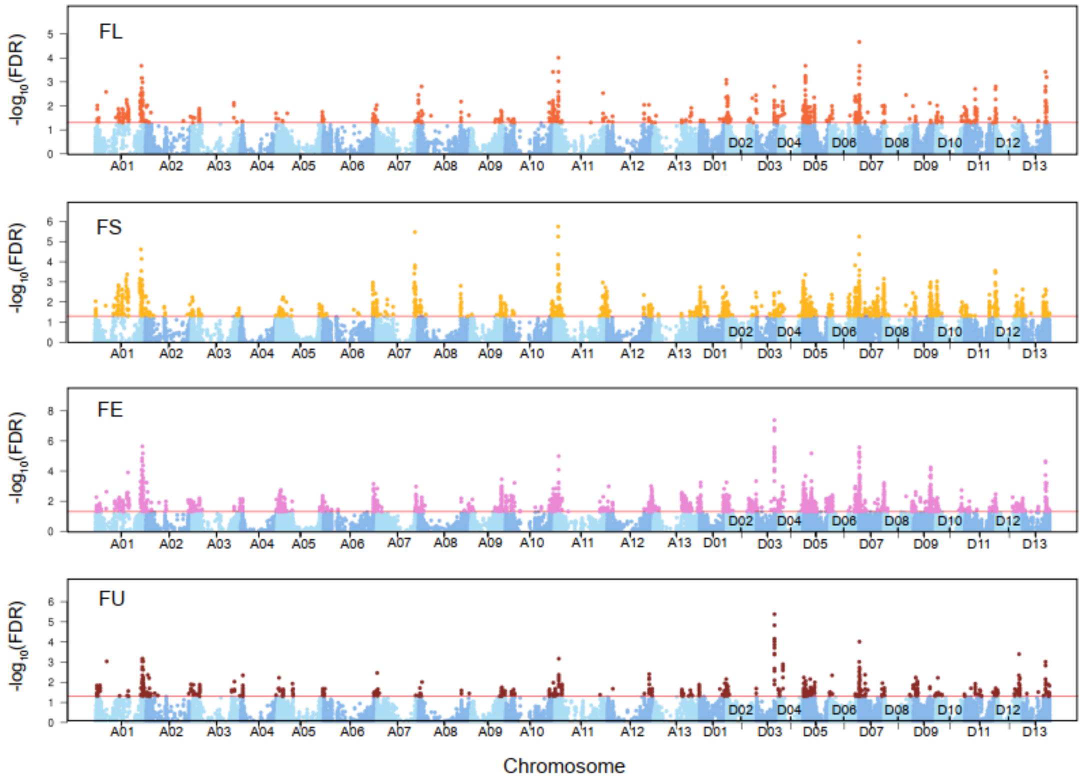

Supplementary Fig. 7: Manhattan plots of the transcriptome-wide association study with four fiber-related traits, respectively. FL, fiber length; FS, fiber strength; FE, fiber elongation rate; FU, fiber uniformity. Significance threshold of FDR = 0.05 (*P*-value of two-sided Student's *t*-test corrected by FDR).

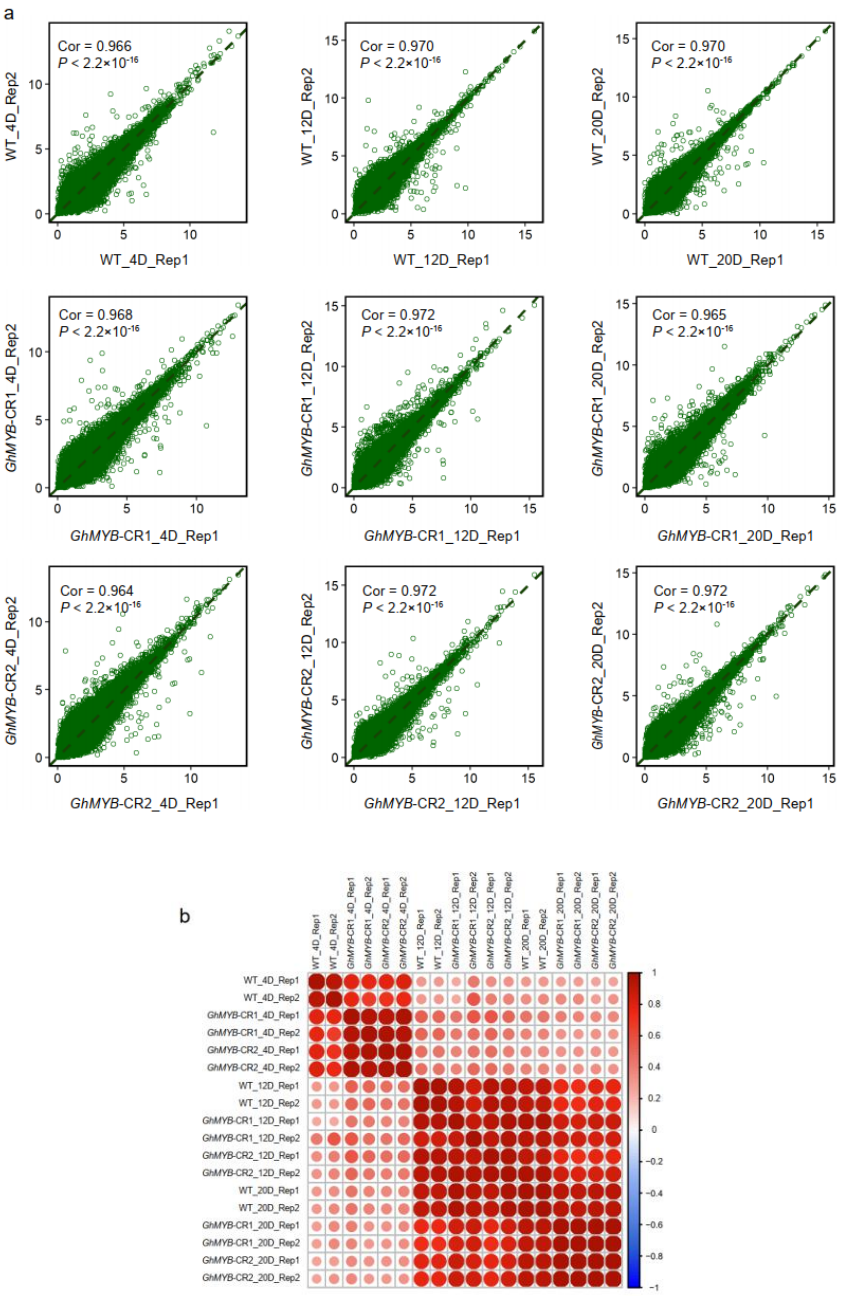

Supplementary Fig. 8: Correlation analysis of gene expression. a, Correlation (Cor) plots of gene expression between two biological replicates (one-sided *F*-test). X-axis: $\log_{10}$(FPKM + 1); Y-axis: $\log_{10}$(FPKM + 1). **b**, Correlation plot of gene expression between different samples and replicates. Correlation coefficients are shown using different colors, where blue represents negative correlation and red represents positive correlation. The size of points represents the absolute values of correlation coefficient.

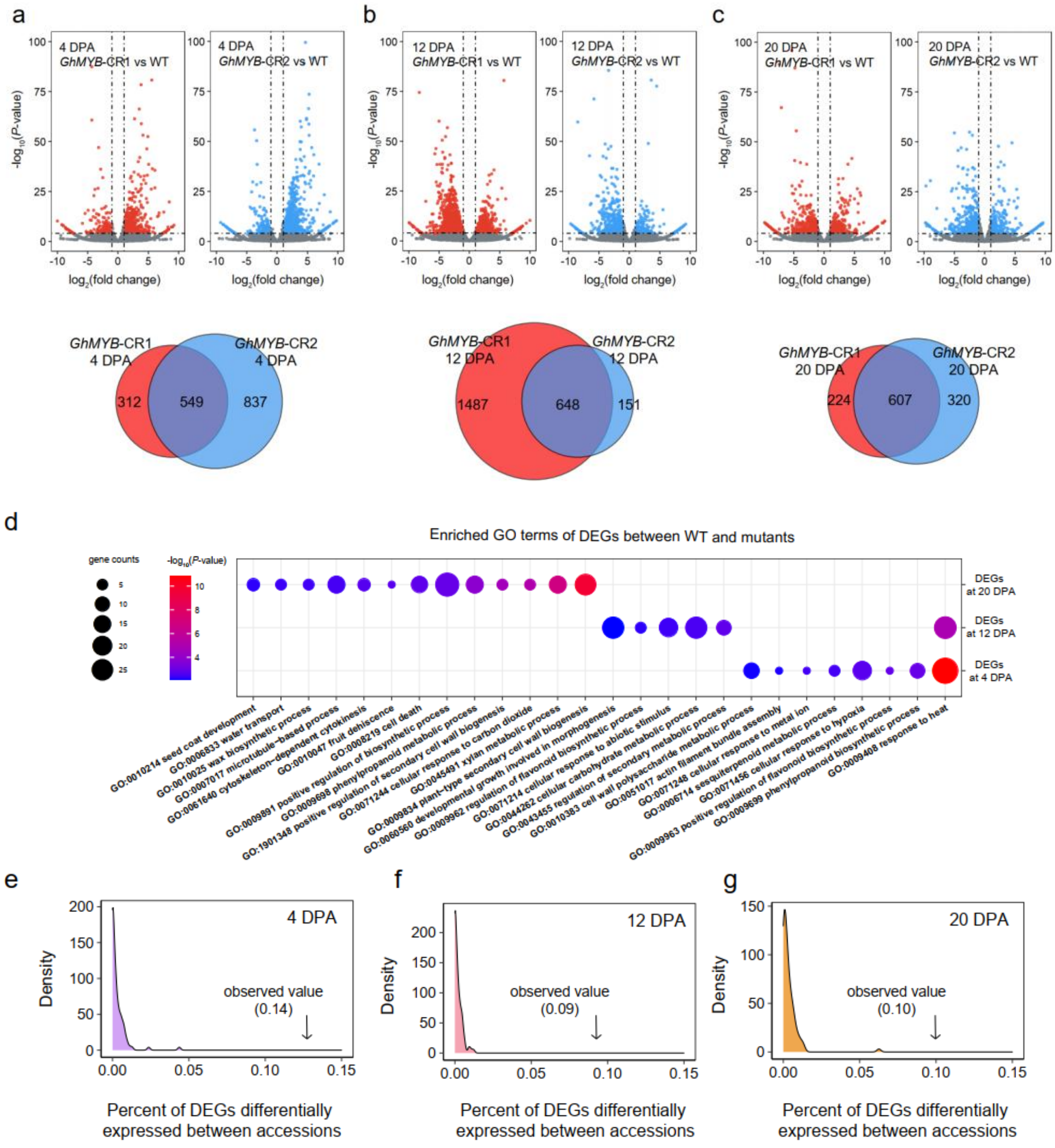

Supplementary Fig. 9: Transcriptome analysis of FL-related TWAS gene mutants. a-c, Up panel: Valcano plots comparing the gene expression of wild type (WT) with *GhMYB* mutants (*GhMYB*-CR1 and *GhMYB*-CR2) in 4 DPA, 12 DPA, 20 DPA fibers, respectively (two-sided Wald test). Dashed lines represent FDR < 0.01 and expression fold change ≥ 2. Differentially expressed genes (DEGs) are marked as red (in *GhMYB*-CR1 vs. WT) and blue (in *GhMYB*-CR2 vs. WT) dots, respectively. Bottom panel: Venn diagram showing DEGs both in *GhMYB*-CR1 vs. WT and *GhMYB*-CR2 vs. WT. **d**, GO enrichment of DEGs between WT and mutants (one-sided Fisher's exact test). **e-f**, The density plots of DEGs differentially expressed between accessions (divided by genotype of *GhMYB* eQTL) in 100 times permutations.

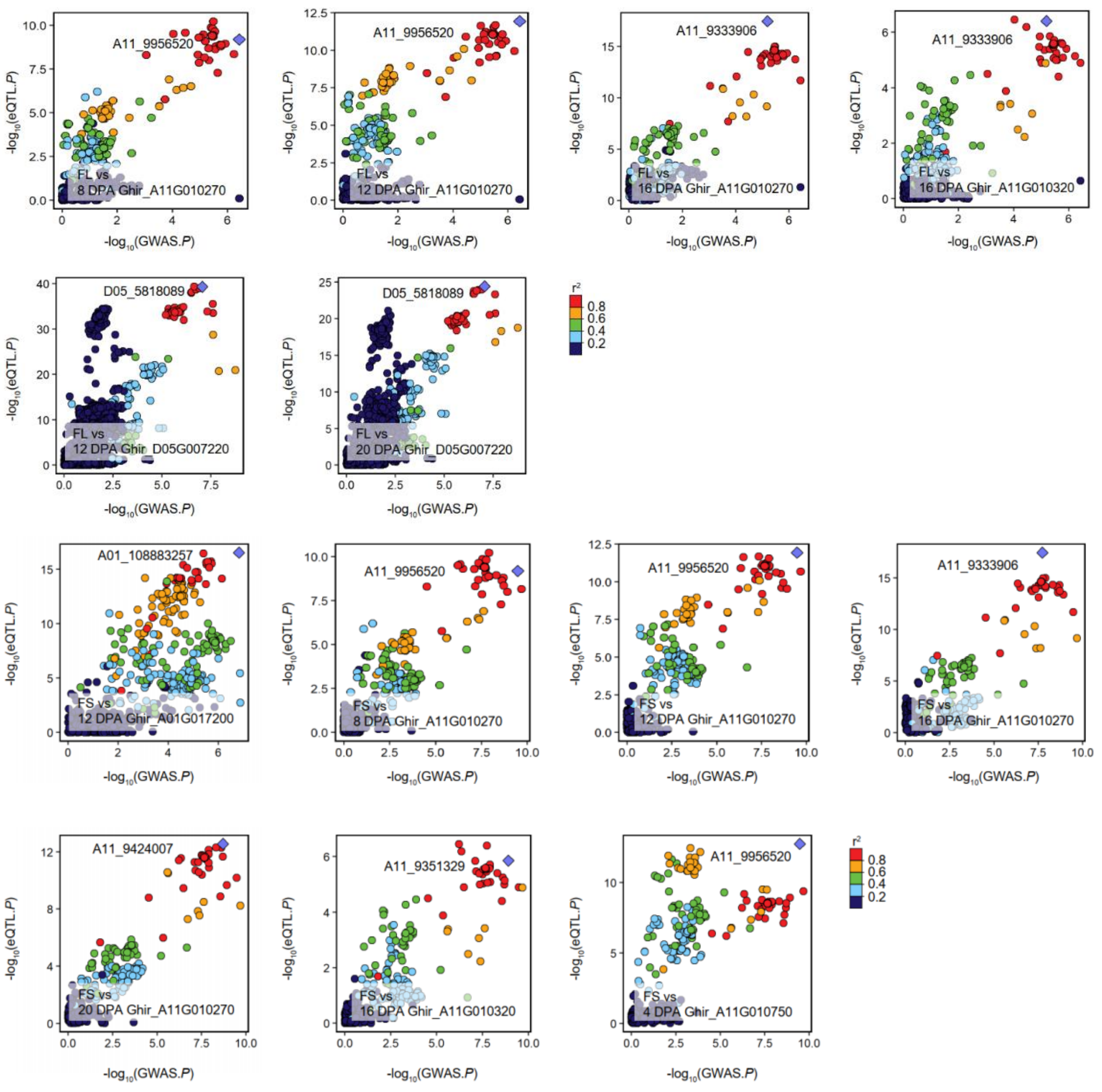

Supplementary Fig. 10: Locus comparison of candidate genes (FL and FS) identified by colocalization. One-sided *F*-test. X-axis: $-\log_{10}$(GWAS.*P*); Y-axis: $-\log_{10}$(eQTL.*P*). Lead SNPs are labeled and highlighted with purple diamonds. Other SNPs are colored with linkage disequilibrium (LD) with lead SNPs.

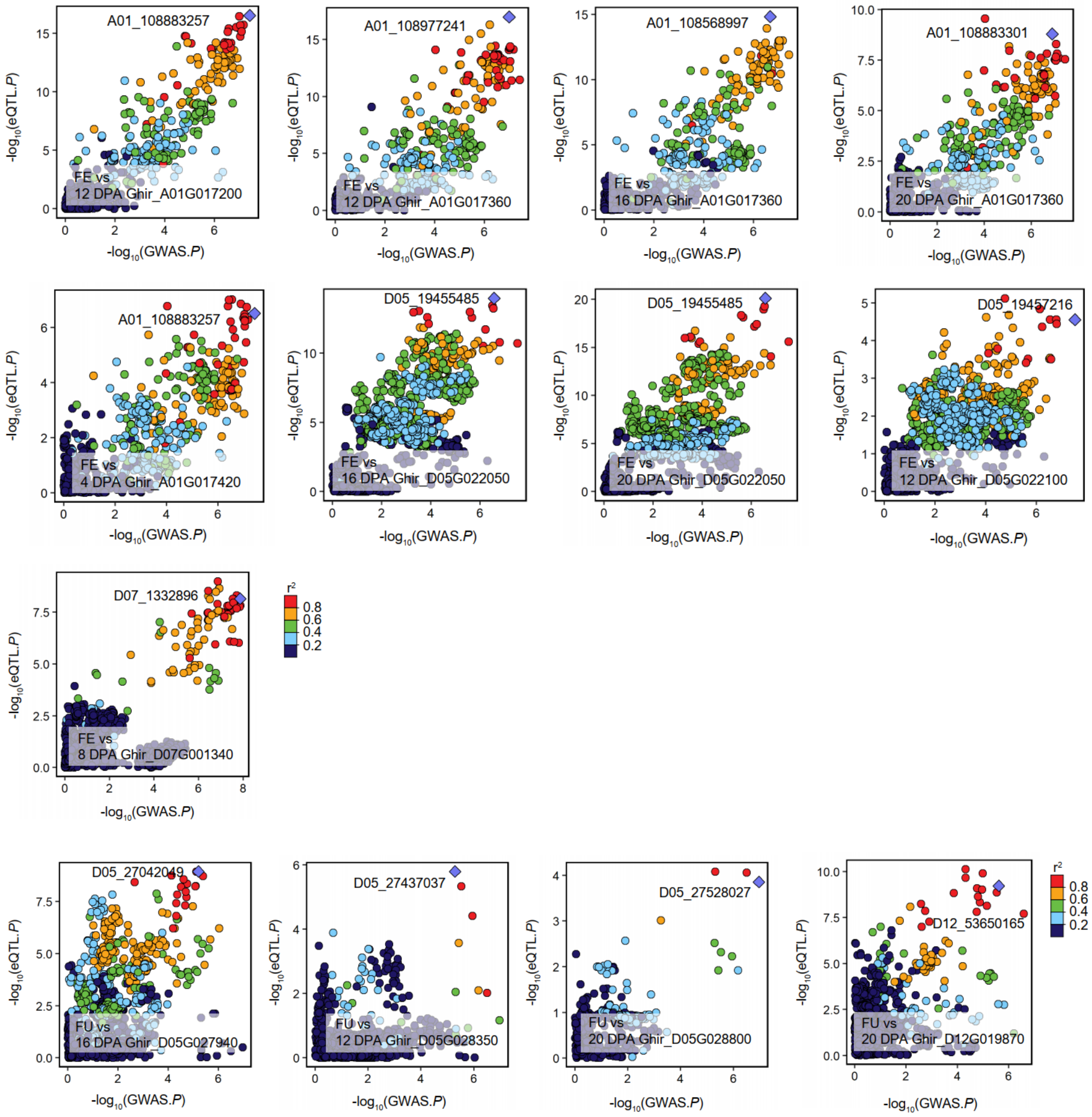

Supplementary Fig. 11: Locus comparison of candidate genes (FE and FU) identified by colocalization. One-sided *F*-test. X-axis: $-\log_{10}$(GWAS.*P*); Y-axis: $-\log_{10}$(eQTL.*P*). Lead SNPs are labeled and highlighted with purple diamonds. Other SNPs are colored with linkage disequilibrium (LD) with lead SNPs.

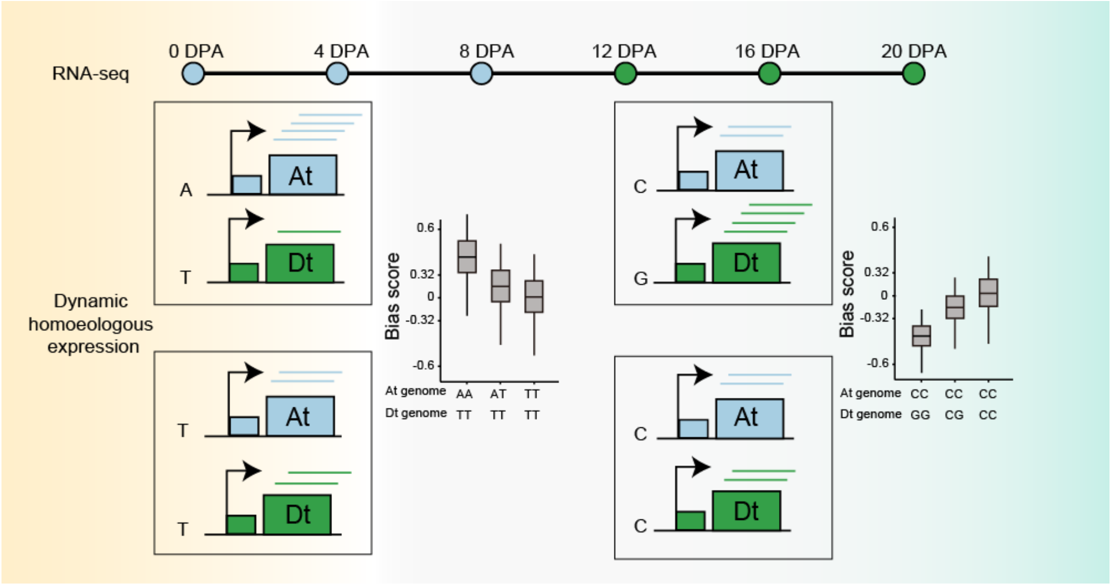

Supplementary Fig. 12: Schematic of bias-eQTL analysis. In this schematic, a homoeologous gene pair showed At-biased expression at early stages (0, 4 and 8 DPA), and showed Dt-biased expression at later stages (12, 16 and 20 DPA). At early stages, the At-subgenomic eQTL with two alleles (A and T), is associated with biased expression of this gene pair. Accessions with A allele in the At subgenome show higher scores of At-biased expression than accessions with T allele, indicating that the eQTL is associated with expression difference between the At and Dt subgenomes. Correspondingly, another eQTL with C and G alleles in the Dt subgenome is linked to expression bias between two subgenomes at later stages. Boxplot elements: center line, median; box limits, first and third quartiles; whisker, 1.5 ×interquartile range.

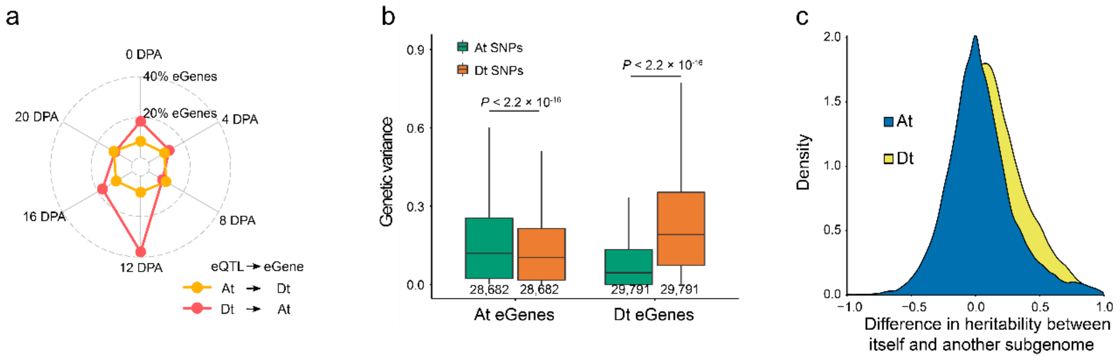

Supplementary Fig. 13: Analysis of genetic regulation across subgenomes. a, The proportion of genes in each subgenome that were regulated by another subgenome. **b**, Genetic variance partitioning for a gene using intra- and inter-subgenomic SNPs (two-sided Wilcoxon rank sum test; center line, median; box limits, first and third quartiles; whisker, 1.5 ×interquartile range). **c**, Comparison of genetic variation components between the At and Dt subgenomes.

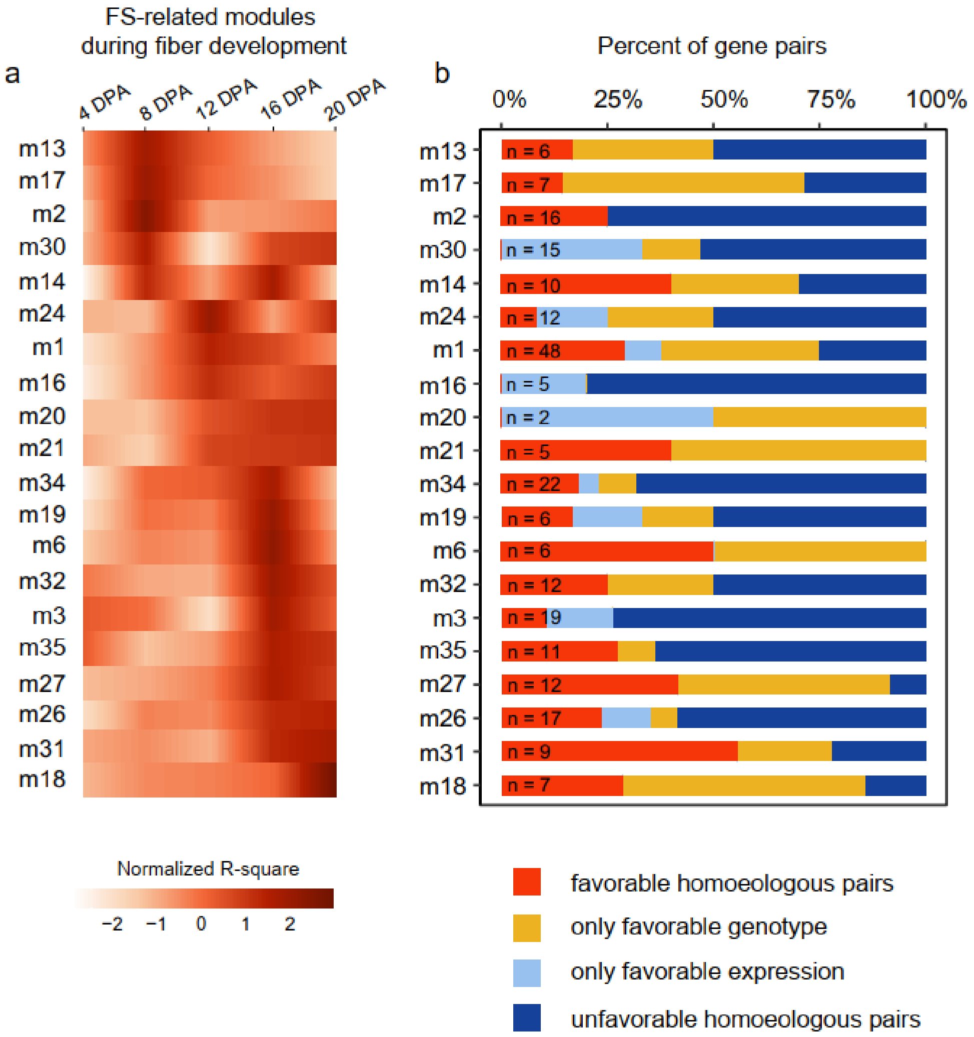

Supplementary Fig. 14: The dynamic interpretation and homoeologous state of FS-related modules. a, Heatmap of dynamic interpretation (Normalized R-square) of fiber strength in 20 modules at 5 timepoints, represented on a white-brown scale. **b**, Stacked bar plot of the states of homoeologous genes in 20 FS-related modules.

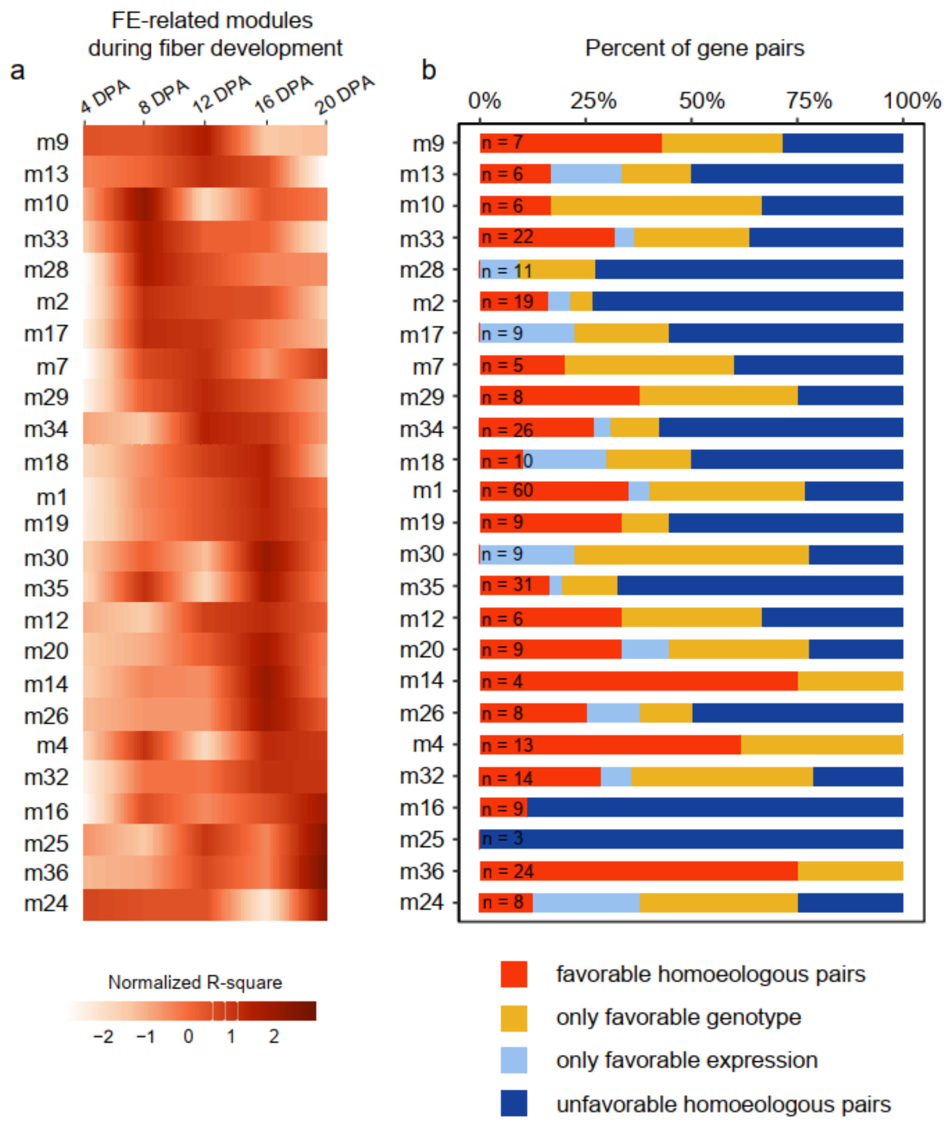

Supplementary Fig. 15: The dynamic interpretation and homoeologous state of FE-related modules. a, Heatmap of dynamic interpretation (Normalized R-square) of fiber elongation rate in 25 modules from 5 timepoints, represented on a white-brown scale. **b**, Stacked bar plot of the states of homoeologous genes in 25 FE-related modules.

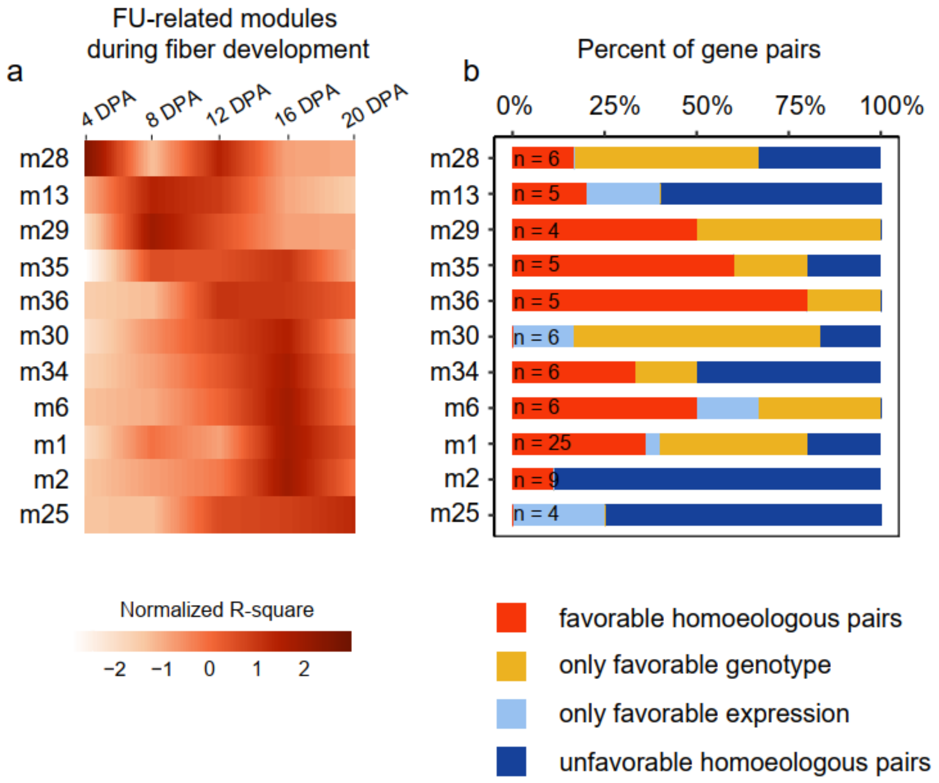

Supplementary Fig. 16: The dynamic interpretation and homoeologous state of FU-related modules. a, Heatmap of dynamic interpretation (Normalized R-square) of fiber uniformity in 11 modules from 5 timepoints, represented on a white-brown scale. **b**, Stacked bar plot of the states of homoeologous genes in 11 FU-related modules.

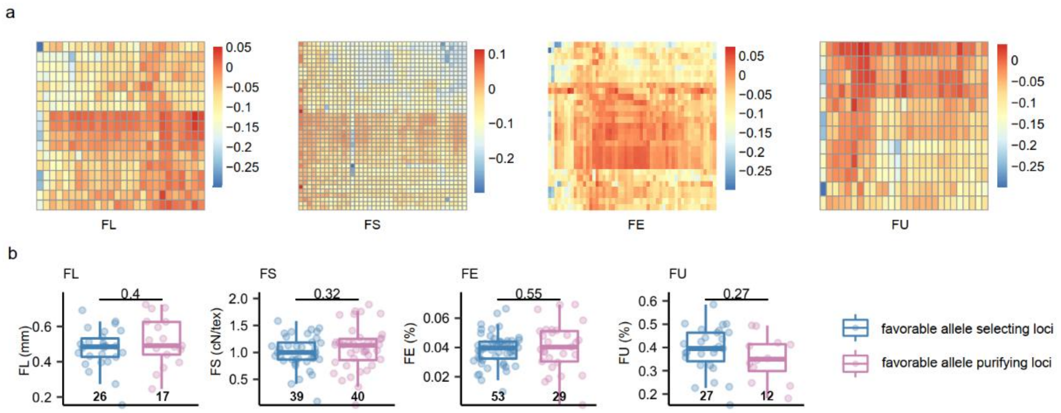

Supplementary Fig. 17: Comparison of two types of loci. **a**, Pearson correlation between the genotype of the two types of loci. Each row represents the loci for which favorable allele was under purifying and each column represents the loci for which favorable allele was under selecting. The genotype was recoded as 0, 1 and 2, representing the dosage of favorable allele. If there's linkage drag, a strong negative correlation should be observed. **b**, Comparison of effect between favorable allele purifying loci and favorable allele selecting loci (two-sided Student's *t*-test; center line, median; box limits, first and third quartiles; whisker, 1.5×interquartile range).

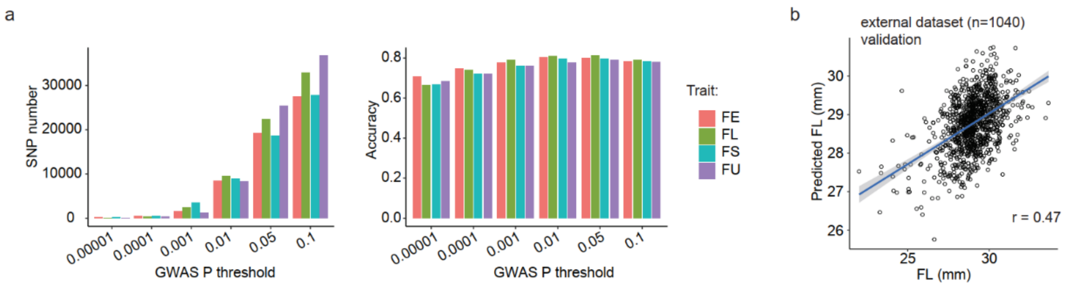

Supplementary Fig. 18: Genomic prediction using rrBLUP. a, Different SNP subsets were used to perform genomic prediction and accuracy was calculated using 5-fold cross-validation repeated 100 times. SNPs in each subset were extracted from all SNPs based on LD-based clumping using PLINK with parameter '--clump-p1' set to different GWAS *P* thresholds. **b**, An external dataset (1,040) were used for testing the accuracy of rrBLUP model for FL. The SNP subset was obtained by using *P* threshold = 0.001 (one-sided *F*-test). The grey band represents 95% confidence interval for the fitted regression.

Supplementary Results

Functional mechanisms of *cis*- and *trans*-eQTLs

We performed enrichment analysis of the lead-eVariants in genomic annotated elements to further understand the molecular mechanism of genetic regulatory effects. As expected, lead-eVariants were significantly enriched in the un-translated regions (UTR) and the primary showed higher enrichment compared to the minor (**Supplementary Fig. 2a**). Compared with randomly selected SNPs, *trans* lead-eVariants show significant enrichment among *cis*-eVariants (LD $\geq$ 0.6, distance $\leq$ 100kb of *cis* lead-eVariants) at the same timepoint (**Supplementary Fig. 2b**), indicating *trans*-eQTLs are partially driven by *cis*-eQTLs.

Analysis of the regulatory components of all eGenes showed that a total of 4,329 eGenes were regulated by both *cis*- and *trans*-eQTL at six timepoints. Among these eGenes, we calculated the LD values (absolute form of genotype correlation) of *cis*- and *trans*-eQTLs that regulate the same gene. At 12 DPA, about 13.1% (492) of *cis*- and *trans*-eQTLs regulating the same eGene showed strong correlation (correlation $\geq$ 0.7) (**Supplementary Fig. 2c**). Across 6 timepoints, *trans* lead-eVariants showed stronger correlation with *cis*-eQTLs than random SNPs with matched minor allele frequency (MAF) (**Supplementary Fig. 2d**).

We also mapped cis-eQTLs by applying stepwise regression to assess whether the identified signal could be attributed to one or more loci. We identified 8,630 independent eQTLs for 4,207 cis-eGenes (**Supplementary Table 5**), of which 10.3%–15.4% had more than one independent cis-eQTL across 6 timepoints. Notably, eVariants with larger effects tended to be closer to the TSS (**Supplementary Fig. 3a,b**). Across 6 timepoints, an average of 25% (range 20.4%–36.1%) of eGenes were detected with more than one eQTL (**Supplementary Fig. 3c**).

GWAS QTL verification using F2 population

To further verify GWAS QTL related to fiber length, we sequenced 200 individuals of an F_2 population from a cross between the long fiber accession (ZY043) and short fiber accession (ZY220). We called SNPs between two parental genomes and genotyped F_2 using re-sequencing data. We identified the regulatory region using

GWAS analysis in the segregating F_2 population. Three significant associations with fiber length were identified on chromosomes D02, D11 (**Supplementary Fig. 6a**). The chromosome D11 association (D11: 18.0 - 24.5 Mb) was colocalized with the locus on chromosome D11 identified by GWAS (qFL-D11) in 376 accessions (**Fig. 2a**). We calculated the SNP index for the long fiber pool (50 individuals) and the short fiber pool (50 individuals) in F_2 population as well as their difference (△SNP index), using 1000-kb sliding window with a step size of 200 kb (**Supplementary Fig. 6b,c**).

***GhMYB* mutants show short fiber phenotypes**

Through TWAS and colocalization analysis of four fiber-related traits, we found a pleiotropic gene *GhMYB* (Ghir_D10G004160) (FS 20 DPA TWAS. FDR = 0.036; FE 12 DPA TWAS. FDR = 0.028) related to the formation of fiber quality. To verify the role of *GhMYB* in fiber development, we used CRISPR/Cas9 system to create *GhMYB* mutants. CRISPR/Cas9 vectors carrying a pair of sgRNA were transferred to the plant JIN668. Through Sanger sequencing, 1 bp deletion event (*GhMYB*-CR1) and 1 bp insertion event (*GhMYB*-CR2) were observed in two mutant lines (**Extended Data Fig. 3a**). In addition, shorter fiber length was observed in both mutant lines compared with wild type JIN668 (**Extended Data Fig. 3b**). These results indicate that *GhMYB* plays a key role in fiber development.

To further evaluate the role of *GhMYB* in fiber development, we sampled the fibers of wild type and mutants at 4, 12 and 20 DPA and performed transcriptome analysis, respectively. Two biological replicates of each sample showed good consistence (**Supplementary Fig. 8a**). The correlation between samples from the same timepoint was higher than correlation between samples from different timepoints (**Supplementary Fig. 8b**). A total of 799 to 2,135 differentially expressed genes (DEGs) were identified between wild-type and mutants at three developmental timepoints **(Supplementary Fig. 9a-c**). At the same timepoint, there were 549 to 648 overlapping DEGs between two different mutant lines, which were enriched in positive regulation of flavonoid biosynthetic process (GO: 0009963), xylan metabolic process (GO: 0045491), plant-type secondary cell wall biosynthesis (GO: 0009834) and other GO pathways related to fiber development (**Supplementary Fig. 9d**).

We next investigated the genotype of eQTL for *GhMYB* (Ghir_D10G004160) in all cotton accessions. About 9-14% of DEGs were differentially expressed between two accession groups which were divided by genotype of *GhMYB* eQTL, and these groups showed significant difference of *GhMYB* expression. Although this proportion (9-14%) was not very high, it was significantly higher than 100 permutations ($P < 0.01$), which suggested genome-wide association analysis of gene expression could reflect real regulatory relationships (**Supplementary Fig. 9e-g**). Among the DEGs, 19, 34, 50 and 23 genes overlapped with TWAS genes were related to FL, FS, FE and FU traits, respectively. These results demonstrate that *GhMYB* plays a key role in fiber development.

Characteristics of genetic modules

In this study, 1,258 candidate genes with corresponding eQTLs/hotspots (including 5 developmental timepoints) constitute fiber quality-related genetic network. After filtering isolated nodes, 813 candidate genes, 1,516 eQTLs, and 68 eQTL hotspots were presented in the genetic network (**Fig. 3c**). According to the built-in program in Gephi (v0.9.5), 2,334 nodes were clustered into 36 modules, and 63 nodes did not belong to any module. Each of the 36 modules include 7–107 candidate genes, 8–183 eQTLs, and 0–10 hotspots (**Fig. 3d**).

For each module, eQTLs corresponding to trait-related candidate genes were used to calculate heritability. We found that 23 FL, 25 FS, 26 FE, and 25 FU-related modules had heritability larger than 0.05 (**Supplementary Table 10**). Based on this threshold, 2 modules (m8 and m23) were associated with single trait, 32 modules (m1, m2, m3, m4, m5, m6, m7, m9, m10, m11, m12, m13, m14, m16, m17, m18, m19, m20, m21, m24, m25, m26, m27, m28, m29, m30, m31, m32, m33, m34, m35, m36) were associated with at least two traits, and the remaining 2 modules (m15 and m22) did not have trait association. Meanwhile, modules with heritability ≥ 0.05 and gene counts ≥ 5 were used for multiple regression analysis of gene expression levels to phenotypic values. The R-square value correlated with the relevance of each module to the phenotype (**Fig. 3e, Supplementary Figs. 14-16 and Supplementary Table 10**).

Genetic regulation across subgenomes

In polyploid cotton, gene expression may be regulated by eQTLs from both the At and Dt subgenomes or either of them. To account for the cumulative effect on gene expression of SNPs from distinct subgenomes, we partitioned genetic regulation for eGenes detected in eQTL analysis. We found that expression variance of genes in the Dt subgenome was mainly explained by intra-subgenomic variants, and more genes in the At subgenome had regulatory variants in the Dt subgenome than Dt had in the At subgenome (**Supplementary Fig. 13a-c**). In addition, we found that there were several hotspots located on the Dt subgenome, which regulated more than 1600 genes on the At subgenome. This may explain why many genes on the At are regulated by eQTLs from the Dt.

Co-expression network and dynamic expression bias of homoeologs

In this study, we found that 16,081 homoeologous gene pairs showed expression bias towards the At (BiasA) or Dt (BiasD) subgenome in at least one timepoint (**Fig. 1d**). Using these gene pairs, we constructed a weighted co-expression network to explore the dynamic expression bias during fiber development (**Extended Data Fig. 5e and Supplementary Table 11**). These 16,081 homoeologous genes were clustered into 16 clusters based on their expression characteristics, and the average relative bias ratio was used to represent the bias trend of each cluster. For example, the cluster 8 shows the pattern of expression bias from BiasN to BiasA during fiber development, which suggests that the expression difference between two homoeologous genes gradually increases (**Extended Data Fig. 5f**). This also suggests a more important role of homoeologous genes from the At subgenome at the latter developmental stages than homoeologous genes from Dt subgenome in cluster 8. The cluster 1 shows the opposite pattern to cluster 8 (**Extended Data Fig. 5g**), and the other clusters exhibit different patterns from clusters 1 and 8, which may reflect the coordinated expression patterns of homoeologous gene pairs during a dynamic developmental process.

To explore the possible genetic regulation relationships for the 16 co-expression clusters, we characterized the homoeologous genes with eQTLs in these clusters. Compared with the three groups that exhibited switched, time-dependent or dominant expression bias during fiber development, we found 7 co-expression clusters had an enrichment of genes from the three groups (**Extended Data Fig. 5h**). Specifically, the

co-expression clusters 1, 7, and 8, with an enrichment of genes from the switched group, show lower network connectivity than those in other co-expression clusters (**Extended Data Fig. 5j**). This suggests that homoeologous genes with dynamic switching of expression bias direction during fiber development have simpler regulatory relationships.

Supplementary Methods

Mutants construction and RNA-seq analysis

The *GhMYB* (Ghir_D10G004160) mutants in cotton were created based on CRISPR technology. Single sgRNA (TCGACTTGAATGACGAAGCT) was designed to target *GhMYB* gene. The primers (*GhMYB*-CR-F: 5'-AAGCATCAGATGGGCAAACAA-AGCACCAGTGGTCTAG-3'; *GhMYB*-CR-R: 5'-TTCTAGCTCTAAAACAGCTT-CGTCATTCAAGTCGATGCACCAGCCGGGAAT-3') were used to amplify tRNA and gRNA from the template pGTR vector. Eventually, the PCR products were purified and inserted the Bsa I-digested pRGEB32-GhU6.9 vector using ClonExpress II One Step Cloning Kit (Vazyme)[1]. The positive vectors were transformed into *Agrobacterium tumefaciens* strain GV3101 for cotton transformation. JIN668 was the transgenic receptor in this study[2].

For each two biological replicates of JIN668 (as wild type) and two *GhMYB* mutants (*GhMYB*-CR1 and *GhMYB*-CR2), total RNA from 4 DPA, 12 DPA, 20 DPA fiber was extracted, sequenced, and transformed to FPKM with the same steps as described in Methods, respectively. The differentially expressed genes between wild type and mutants were identified by DESeq2 software (v1.24.0)[3], respectively.

Annotation of functional variants

We analyzed the enrichment of eVariants in functional elements. ANNOVAR[4] was run to determine if SNPs caused changes in protein-coding regions or were located in specific genomic regions. Transcript factor binding site (TFBS) data were predicted in the P-Match[5] corresponding plant database using the promoter sequence of the gene upstream of 2 kb. Within each timepoint, we grouped all eVariants for each target gene into categories with the most significant (primary) and minor ones (minor), and then compared them to the random selection of variants in the 1 Mb region of each timepoint, respectively.

Identification of favorable expression pattern

To identify favorable expression pattern for each candidate gene, 340 accessions sampled in 5 fiber developmental timepoints were retained for further analysis. The

normalized expression of 1,258 candidate genes × 340 accessions in 5 fiber developmental timepoints were clustered into 12 groups by k-means (**Extended Data Fig. 3d**). The K = 12 was defined by Elbow method[6]. For each candidate gene, 340 accessions were clustered into 1-12 groups (expression patterns). We identified favorable expression pattern with following steps:

1) Filtering genes. Only genes with more than 1 expression pattern in 340 accessions were retained. 2) Filtering expression patterns. For each retained gene, only expression patterns corresponding to at least 3 accessions were retained. 3) Significance test. For each retained gene, a significance analysis (wilcox.test) of phenotypic values was performed between accessions corresponding to different expression patterns. The P-value < 0.05 indicates that there were significant differences between the two expression patterns.

For each candidate gene, the expression patterns corresponding to the accessions with significantly higher phenotypic values than other patterns were identified as "favorable expression pattern".

Identification of co-expression clusters with dynamic expression bias

We constructed a co-expression network of the 16,081 homoeologous genes using WGCNA (Weighted correlation network analysis) (v1.70.3)[7]. By combining the expression matrix of a pair of homoeologous gene in 2,215 samples (376 accessions and 6 timepoints), we used 4,430 FPKM values to represent the expression characteristics of this gene pair in 376 accessions. Different values for "power" were found (range from 1 to 30). As the degree of independence reached 0.9, 5 was determined as the most appropriate "power". Homoeologous genes were divided into 17 co-expression clusters. In order to reduce the computational complexity, edges with weight < 0.18 were filtered out. Finally, 13,253 homoeologous gene pairs in 16 co-expression clusters were retained to represent the network of homoeologous genes.

In order to quantify the characteristics of expression bias for each gene pair, we proposed the relative bias ratio:

$$\text{Relative bias ratio} = (percentage\ of\ BiasA - percentage\ of\ BiasD) \times (percentage\ of\ BiasA + percentage\ of\ BiasD)$$

The relative bias ratio ranges from -1 to 1, which shows the closer to 1, more accessions showed expression bias towards the At subgenome for each homoeologous gene pair; the closer to -1, more accessions showed expression bias towards the Dt subgenome. For each cluster, the average relative bias ratio of all genes represented the bias level of this cluster. The closer to 1, the more gene pairs in the cluster showed expression bias towards the At subgenome; the closer to -1, the more gene pairs in the cluster showed expression bias towards the Dt subgenome. The average relative bias ratio of 16 clusters calculated at 6 timepoints could reflect the dynamic bias of homoeologous gene pairs.

Identification of pseudo-regulatory sites in the other subgenome

In order to analyze the relationship between regulatory sequence differences and expression bias of homoeologous genes, only *cis*-eQTL affecting homoeologous gene expression bias were used for analysis. For each eQTL, we identified the genomic coordinates of all LD-friend SNPs in another subgenome using the pure-python implementation of UCSC liftOver program (pyliftover v0.4). Specifically, we extracted a 200 bp sequence of SNP upstream and downstream. For each sequence, we aligned it to the 2 Mb region upstream and downstream of TSS of the homoeologous gene. Alignments were generated using LASTZ (v1.04.03) with following parameters: K = 3000 L = 3000 H = 2000 Y = 5000 E = 55 T = 2 O = 600 --filter = identity:80 --filter = coverage:85 --verbosity = 10 --format = axt (http://www.bx.psu.edu/~rsharris/lastz/). Alignments were transformed into chain files using several University of California, Santa Cruz (UCSC) tools[8]. Briefly, the axtChain UCSC tool was run with parameters: -minScore = 3000 -linearGap = medium. Then PyLiftover was used to convert genomic coordinates between subgenomes, and matches with the highest scores were retained. Among 860,433 LD-friend SNPs, 407,708 query sequences were conserved between two subgenomes. For each SNP, we calculated a score according to whether its genotype was different in the two subgenomes. Briefly, in each sample, the score of SNP was set for 1 when its sequence is identical between two subgenomes, -1 when it is not, and 0 when it is not conserved. For all LD-friend SNPs of each bias-eQTL, the cumulative score was calculated. The higher cumulative score in a sample indicates the more similar bias-eQTL between two subgenomes.

Genotype and GWAS analysis for F_2 population

In addition to the 376 accessions sequenced for GWAS analysis, an F_2 population with 1,809 individuals from a cross between long fiber accession (S309, ~31 mm) and short fiber accession (S304, ~25 mm) was constructed, of which 200 were genotyped using DNA resequencing. An average of 8.4 Gb data was generated for each individual and SNP calling was described in Methods. We filtered SNP with missing rate (≤ 20%) and used beagle (v4.1)[9] to impute the missing genotype of each individual. A total of 637,372 high quality SNPs were identified between two parental genomes and F2 population. With genotype data, we performed GWAS analysis using phenotype from 200 individuals and calculated the SNP index for the long fiber pool (50 individuals) and the short fiber pool (50 individuals) in F_2 population as well as their difference (△SNP index), using 1,000-kb sliding window with a step size of 200 kb.

Phylogenetic analysis

To explore the phylogenetic relationship among the 3,552 cotton accessions, a subset of 300,000 SNPs was randomly extracted and the script vcf2phylip (https://github.com/edgardomortiz/vcf2phylip) was used to convert SNPs in VCF format to FASTA alignments. FastTree was used to infer a phylogenetic tree and R package ggtree (v2.4.2) was used to plot the tree[10].

Construction of favorable allele library

We collected DNA sequencing data for a total of 3,552 accessions, of which 3,181 were from public data[11-14]. Adaptors and low-quality reads were filtered using fastp (v0.23.0)[15] and then Sentieon DNASeq variant calling workflow was used for variant calling (https://github.com/Sentieon/sentieon-dnaseq). In brief, clean reads were aligned to the *G. hirsutum* reference genome (TM-1)[16] using 'sentieon bwa mem' command[17]. Cram files were indexed and sorted using SAMtools (v1.9)[18]. PCR duplicate reads were removed by the 'sentieon driver --algo Dedup --rmdup' command. Haplotyper algorithm (sentieon driver --algo Haplotyper) was used to perform variant calling with parameter '--genotype_model multinomial --emit_mode gvcf'. We extracted the trait-associated loci from each gvcf file using Python package

pysam (v0.16.0.1) (https://pysam.readthedocs.io/en/latest/api.html). GVCFtyper algorithm (sentieon driver --algo GVCFtyper) was used to perform the joint calling. The genotype in each locus was recoded as 0, 1 and 2, which represented the dosage of the favorable allele.

References

1. Wang, P., *et al*. High efficient multisites genome editing in allotetraploid cotton (*Gossypium hirsutum*) using CRISPR/Cas9 system. *Plant Biotechnol. J.* **16**, 137–150 (2018).

2. Sun, L. *et al.* Red fluorescent protein (DsRed2), an ideal reporter for cotton genetic transformation and molecular breeding. *Crop J.* **6**, 48–58 (2018).

3. Anders, S., & Huber, W. Differential expression analysis for sequence count data. *Genome Biol.* **11**, R106 (2010).

4. Wang, K., Li, M., & Hakonarson, H. ANNOVAR: functional annotation of genetic variants from high-throughput sequencing data. *Nucleic Acids Res.* **38**, e164 (2010).

5. Chekmenev, D. S., Haid, C., & Kel, A. E. P-Match: transcription factor binding site search by combining patterns and weight matrices. *Nucleic Acids Res.* **33**, W432–W437 (2005).

6. Liu, F & Deng, Y. Determine the Number of Unknown Targets in Open World Based on Elbow Method. *IEEE Trans. Fuzzy Syst.* **29**, 986–995 (2021).

7. Langfelder, P. & Horvath, S. WGCNA: an R package for weighted correlation network analysis. *BMC Bioinformatics* **9**, 559 (2008).

8. Kuhn, R. M., Haussler, D. & Kent, W. J. The UCSC genome browser and associated tools. *Brief. Bioinform.* **14**, 144-161 (2013).

9. Browning, B. L., Tian, X., Zhou, Y., & Browning, S. R. Fast two-stage phasing of large-scale sequence data. *Am. J. Hum. Genet.* **108**, 1880–1890 (2021).

10. Yu, G. C., Smith, D. K., Zhu, H. C., Guan, Y. & Lam, T. T. Y. GGTREE: an R package for visualization and annotation of phylogenetic trees with their covariates and other associated data. *Methods Ecol. Evol.* **8**, 28-36 (2017).

11. Wang, M. *et al.* Asymmetric subgenome selection and cis-regulatory divergence during cotton domestication. *Nat. Genet.* **49**, 579–587 (2017).

12. Ma, Z. *et al.* High-quality genome assembly and resequencing of modern cotton cultivars provide resources for crop improvement. *Nat. Genet.* **53**, 1385–1391 (2021).

13. He, S. *et al.* The genomic basis of geographic differentiation and fiber improvement in cultivated cotton. *Nat. Genet.* **53**, 916–924 (2021).

14. Yuan, D. *et al.* Parallel and Intertwining Threads of Domestication in Allopolyploid Cotton. *Adv. Sci.* **8**, 2003634 (2021).

15. Chen, S., Zhou, Y., Chen, Y. & Gu, J. fastp: an ultra-fast all-in-one FASTQ preprocessor. *Bioinformatics* **34**, i884–i890 (2018).

16. Wang, M. et al. Reference genome sequences of two cultivated allotetraploid cottons, *Gossypium hirsutum* and *Gossypium barbadense*. *Nat. Genet.* **51**, 224–229 (2019).

17. Li, H. & Durbin, R. Fast and accurate short read alignment with Burrows–Wheeler transform. *Bioinformatics* **25**, 1754–1760 (2009).

18. Li, H. *et al.* The Sequence Alignment/Map format and SAMtools. *Bioinformatics* **25**, 2078–2079 (2009).
